# Overexpression of aquaporin 4 in articular chondrocytes exacerbates the severity of adjuvant-induced arthritis in rats: an *in vivo* and *in vitro* study

**DOI:** 10.1186/s12950-017-0153-8

**Published:** 2017-03-01

**Authors:** Li Cai, Chao Lei, Rong Li, Wei-na Chen, Cheng-mu Hu, Xiao-yu Chen, Chun-mei Li

**Affiliations:** 1 Department of Pathology, School of Basic Medicine, Anhui Medical University, 81 Meishan Road, Hefei, 230032 Anhui Province China; 2 School of Pharmacy, Anhui Medical University, 81 Meishan Road, Hefei, 230032 Anhui Province China; 3 Department of Histology and Embryology, School of Basic Medicine, Anhui Medical University, 81 Meishan Road, Hefei, 230032 Anhui Province China

**Keywords:** Aquaporin 4, Adjuvant-induced arthritis, Articular cartilage, Articular chondrocytes, Rheumatoid arthritis

## Abstract

**Background:**

The dysfunction of articular chondrocytes is a crucial step in rheumatoid arthritis (RA) pathogenesis while its molecular mechanisms are not fully known. This study was aimed to investigate the expression of aquaporin 4 (AQP4) in articular chondrocytes of adjuvant-induced arthritis (AIA) rats and its involvement in AIA development.

**Methods:**

Thirty rats were divided into normal and AIA group (*n* = 15). Rat AIA was induced by intradermal injection of complete Freund’s adjuvant and evaluated by secondary paw swelling and histological assessments on knee joint damage. Localization and protein expression of AQP4 in articular cartilage were examined by immunohistochemistry and western blot. In vitro study, AIA articular chondrocytes were cultured and treated with acetazolamide, an AQPs inhibitor. AQP4 protein level, cell proliferation and mRNA levels of type-II collagen (COII) and aggrecan were measured by western blot, MTT assay and real-time PCR, respectively.

**Results:**

The results of immunohistochemistry and western blot indicated that AQP4 showed higher protein levels in cartilage tissues of AIA rats than that of normal rats. Correlation analysis revealed that AQP4 protein level in cartilage tissues of AIA rats remarkably correlated positively with secondary paw swelling on day 26 after AIA induction as well as pathological scores on joint damage. Additionally, acetazolamide treatment effectively decreased AQP4 protein level, increased cell proliferation and mRNA levels of COII and aggrecan, suggesting AQP4 inhibition by acetazolamide could normalize the dysfunction of AIA articular chondrocytes in vitro.

**Conclusions:**

Our data provide certain experimental evidence that AQP4 over-expression in articular chondrocytes aggravated AIA severity and might be a novel target for RA treatment.

## Background

Rheumatoid arthritis (RA) is a chronic autoimmune inflammatory disease and usually causes serious disability in affected individuals [1]. Joint swelling, synovial inflammation and progressive destruction of cartilage are the hallmarks of RA [2]. Previous studies on RA pathogenesis predominantly focus on synovial inflammation and excessive synovial hyperplasia. However, accumulating clinical evidence reveal that the joint destruction is not always correlated with inflammatory process in RA [3], joint destruction still occurs even when the joint inflammation is suppressed by proper anti-arthritic treatment [4]. Particularly, articular cartilage damage is positively associated with irreversible RA disability, and more attention should be taken to the molecular pathomechanism of articular cartilage damage in RA [5].

As the unique cellular component in cartilage, articular chondrocytes are responsible for synthesizing and maintaining the cartilage extracellular matrix (ECM) which mainly consists of water, type-II collagen (COII) and proteoglycans [6]. The metabolic activity of articular chondrocytes is influenced by local extracellular physicochemical factors including pH, fluid flow, ionic and osmotic environment [7]. In the affected joints of RA, multiple extracellular pathological changes such as hydrarthrosis, acidosis and hyperosmotic stress induce the dysfunction of articular chondrocytes, e.g., excessive apoptosis and reduced ECM synthesis ability, eventually lead to articular cartilage destruction [8].

Aquaporins (AQPs) belong to membrane transport proteins family and are closely involved in regulating the influx and outflow of water and small molecules [9]. At least 13 mammalian AQPs (AQP0-12) have been studied in various tissues and organs including kidney, eye, brain and lung [10]. The permeability of AQPs is dependant on the osmotic and hydrostatic gradients and pH values [9, 11]. Several investigations have showed underlying involvement of AQPs in cartilage damage of joint diseases such as RA and osteoarthritis (OA). Mobasheri et al. firstly confirmed that AQP1, 3 are expressed in equine articular cartilage [12]. AQP1 expression was found significantly increased in articular cartilage of RA [13, 14] and OA patients or experimentally induced OA rats [15, 16]. Nagahara et al. observed that AQP9 was strongly expressed in the synovial tissues of OA and RA patients, and was related to the pathogenesis of hydrarthrosis and synovitis [17]. It is well known that AQP4 possess very high single channel water permeability (3-fold greater than that of AQP1) [18], while previous studies of AQP4 mainly aim at nervous system diseases [19]. There is high homology between human and rat AQP4 (93%). Interestingly, our recent work suggested that AQP4 expressed in human cartilage tissues. However, the potential pathological role of AQP4 in RA cartilage damage is still unknown.

In this study, rat with adjuvant-induced arthritis (AIA) was established and used as an experimental model resembling RA. We investigated the AQP4 expression in articular cartilage of AIA rats and revealed potential involvement of AQP4 activation in the development of rats AIA. Acetazolamide is a widely used sulfonamide carbonic anhydrase inhibitor and is recently shown to be a potent inhibitor of AQPs [20, 21]. In vitro studies, we observed the effects of acetazolamide on AQP4 expression, cell proliferation and mRNA levels of COII and aggrecan in cultured AIA chondrocytes.

## Methods

### Animals and AIA induction

Male Sprague Dawley rats weighting 140–160 g were purchased from Experimental Animal Center of Anhui Medical University and housed under standardized conditions of temperature (20–22 °C) and humidity (50–60%). After 7-day acclimatization, the rats were randomly divided into normal rats group and AIA experimental group (15 rats per group). Commercial complete Freund’s adjuvant (CFA) (Catalog #7027, 10 mg/mL) was got from Chondrex, Inc (WA, USA). Each Rat in AIA experimental group received a single intradermal injection of 0.1 mL CFA into the right hind paw to induce arthritis [22]. Meanwhile, the equivalent volume of saline was given to each rat in normal group.

### Evaluation of arthritis development

The day of CFA injection was regarded as day 0. The volume of the non-injected (left) hind paw was measured by a plethysmograph apparatus (YLS-7A, Academy of Medical Science of Shandong Province, China) at various time points including day 0 and day 10, 14, 18, 22, 26 after AIA induction. The secondary paw swelling at each time point was defined as the changes in paw volume on day 0 (basic value) and day 10, 14, 18, 22, 26 (ΔmL).

### Histopathological examination and assessment of joint damage

Animals were euthanized on day 26 and then the left knee joints were quickly removed, trimmed, fixed in 4% paraformaldehyde. After decalcification in 10% EDTA for 2 weeks, the tissues were paraffin embedded and sliced at 5 μm thick for histopathological examination. The paraffin sections with hematoxylin and eosin (HE) staining were observed under a light microscope (Nikon 80i, Japan) and evaluated by two trained pathologist blinded to the specimens. Pathological assessments of joint damage were carried out on the basis of synovial hyperplasia, cartilage destruction, vascular proliferation and inflammatory cells infiltrate. Pathological scores on the severity of lesions were classified into four grades: 0, no detectable changes; 1, mild; 2, moderate; 3, severe. The average of three slides per rat was used as an independent data for statistical analysis. Total pathological scores of joint damage were the sum of the individual score for every pathological index.

### AQP4 immunohistochemistry staining and semi-quantitative analysis

Immunohistochemistry staining for AQP4 was performed according to standard procedures [23]. The knee joint sections were hydrated, rinsed and microwave-treated in 0.05 M citrate-buffered saline. Sections were treated by 3% hydrogen peroxide in methanol at room temperature for 10 min to quench endogenous peroxidase activity and incubated in 5% goat serum at 37 °C for 15 min to block nonspecific staining. Then sections were incubated at 4 °C overnight with primary antibody of AQP4 (1:100; Abcam plc, Cambridge, UK; Catalog ab9512). The section of knee joint without adding AQP4 antibody was applied as a negative control. Sections were incubated with biotinylated secondary antibodies and avidin-biotin horseradish peroxidase complex (1:200; Vector Laboratories, CA, USA) at 37 °C for 30 min. Sections were visualized by diaminobenzidine, dehydrated, hyalinized in xylene and mounted with neutral gum. Finally, sections were observed by a light microscope and typical photos were taken. Cell membrane and cytoplasm contained yellow/brown granules were considered positively stained cells. AQP4 staining scores were determined by semi-quantitative optical analysis. The number of positive cells and total cells were counted at five fields per slide. We calculated the positive expression rate (%), which was defined as (number of positive cells/total cells) × 100%. The proportion of positive cells was graded as follows [16]: (−), < 5%; (+), 5–25%; (++), 26–50%; (+++), 51–75%; (++++), > 75%. The average of three slides was used as an independent data for statistical analysis.

### Western blot analysis

Aliquots of cartilage tissues obtained from the right knee joints were homogenized and treated with RIPA lysis buffer. The protein levels of lysates were measured by Bradford assay. An equal weight of protein was separated by SDS-PAGE and transferred onto a nitrocellulose membrane. The membranes were blocked with 5% skim milk and incubated with the primary antibodies against AQP4 (1:500) or β-actin (1:500) at 4 °C overnight. Subsequently, the membranes were incubated with a horseradish peroxidase conjugated secondary antibody at 37 °C for 2 h. The protein bands were easily visualized using Enhanced Chemiluminescence Kit (Pierce, IL, USA). Then the bands were scanned and measured by ImageJ software. The ratio of optical density value of interest protein band over its corresponding β-actin band was recognized as the relative protein level.

### Articular chondrocytes preparation and acetazolamide treatment

Another 10 AIA rats and 10 normal rats were prepared for the in vitro experiments. Rats were euthanized and cartilage tissues from knee joints were quickly collected. Articular chondrocytes were isolated and cultured similar to previously described [7, 24]. Briefly, small minced cartilage tissues (about 1 mm^3^) was digested with 0.25% trypsin for 0.5 h and 0.2% type II collagenase for 3 h in cell incubator, respectively. The isolated cells were pipetted through 200-mesh nylon mesh into centrifuge tubes and washed with PBS. Then the freshly isolated chondrocytes were resuspended in DMEM medium (Thermo Fisher Scientific, PA, USA) containing 10% fetal calf serum (FCS), penicillin (100 IU/ml) and streptomycin (100 μg/ml) and incubated in a flat-bottomed culture bottle at 37 °C, 5% CO_2_ for 5 days. Adherent cells were trypsinized, split and recultured in DMEM medium. The articular chondrocytes of 1–2 passages were applied in this study. The cultured chondrocytes were identified by the morphological property and toluidine blue staining of glycosaminoglycan. The cultured articular chondrocytes were divided into 4 groups: normal articular chondrocytes, AIA articular chondrocytes and AIA articular chondrocytes treated with acetazolamide (25 or 100 μM). Acetazolamide (Sigma, MO, USA) was dissolved with DMSO to prepare the stock solution (100 mM) and diluted to the final concentrations using DMEM medium containing 10% FCS.

### AQP4 expression in cultured articular chondrocytes

AQP4 immunocytochemistry staining was performed in cultured normal and AIA chondrocytes, and the detailed procedure of immunocytochemistry was similar to the immunohistochemical steps described above. In addition, we carried out western blot analysis for AQP4 in various cultured chondrocytes groups to investigate whether acetazolamide treatment inhibited the protein levels of AQP4 in AIA chondrocytes.

### Chondrocytes proliferation measurement

Articular chondrocytes were prepared and suspended in DMEM medium containing 10% FCS at a concentration of 5 × 10^7^ cells/L. 100 μL cell suspension was added to 96-well plate and incubated at 37 °C, 5% CO_2_ for 24 h. After adherence, the culture medium was replaced by 200 μL DMEM or DMEM with different doses of acetazolamide for 48 h cultivation. 10 μL of MTT (Sigma, MO, USA) (5 g/L) was added to each well and the cells were incubated for another 4 h. After centrifugation, the supernatants were removed. The formazan crystals derived from MTT were dissolved completely with 150 μL dimethyl sulfoxide per well. The absorbance (A) was measured at 570 nm using a microplate reader and the results were described as the average of A.

### Real-Time quantitative PCR (Q-PCR) of COII and aggrecan mRNA

Total RNA was extracted from articular chondrocytes in various groups by Trizol method (Invitrogen, CA, USA). cDNA were synthesized by a RevertAid First Strand cDNA Synthesis Kit (Thermo Scientific, PA, USA). Q-PCR was performed by SYBR Green PCR Kit (Applied Biosystems, USA) in ABI Prism 7000 Sequence Detector. Cycling conditions in 25 μL volume were as follows: 15 s at 95 °C, 60 s at 64 °C (COII) or 60 °C (aggrecan); 40 cycles. PCR primers were purchased from Sangon Biotech Company (Shanghai, China): COII 5’-TCAAGTCGCTGAACAACCAG-3’ and 5’-GTCTCCGCTCTTCCACTCTG-3’; Aggrecan 5’-GCAGCACAGACACTTCAGGA-3’ and 5’-CCCACTTTCTACAGGCAAGC-3’; β-actin 5’-TTGCTGACAGGATGCAGAA-3’ and 5’-ACCAATCCACACAGAGTACTT-3’. The relative amplification efficiencies of the primers were tested and shown to be similar. 2^-ΔΔCt^ method was used to measure the relative amount of mRNA and the result was normalized against β-actin.

### Statistical analysis

Statistical analysis was carried out by SPSS 16.0 software. The experimental data were analyzed by Independent-Samples *T* test or One-Way ANOVA followed by LSD post hoc test. All values were shown as mean ± standard error of the mean (SEM) and *p* < 0.05 was considered to be statistically significant. Correlation between AQP4 protein levels of AIA rats and secondary hind paw swelling or pathological scores on joint damage was determined by Pearson’s correlation test.

## Results

### Evaluation of AIA in rats

**Fig. 1:**
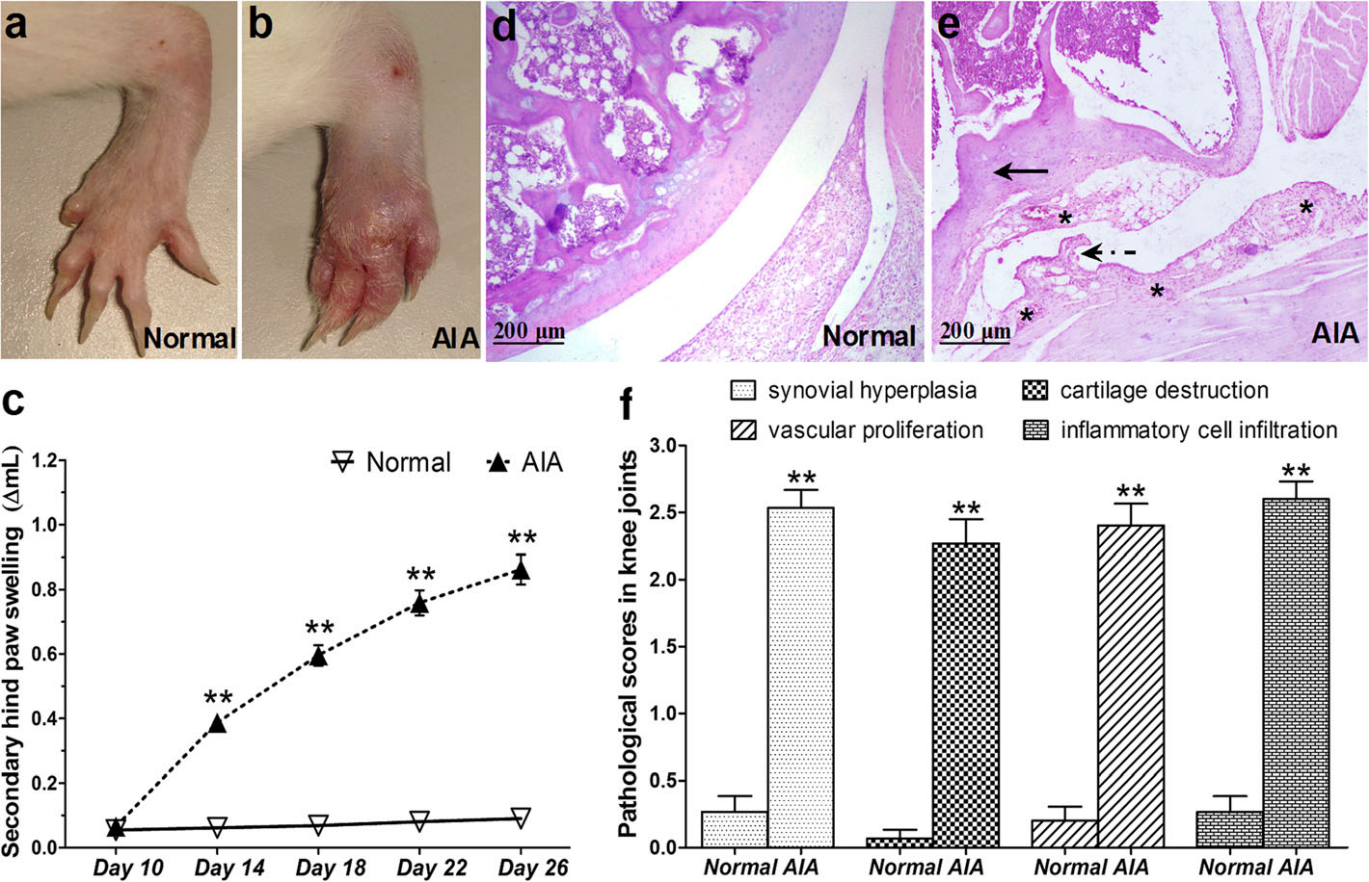
Evaluation of AIA in rats. **a**, **b** Representative photos of non-injected (*left*) hind paw on day 26 after induction (**a**, Normal; **b**, AIA). **c** The changes of secondary hind paw swelling on different time points. **d**, **e** Typical histopathological photos of knee joint sections with HE staining (×100; **d**, Normal; **e**, AIA). In the HE fig. of knee joint from AIA rats, typical pathological characteristics resembling RA including synovial hyperplasia (*dotted arrow*), cartilage damage (*arrow*), vascular proliferation (*) and excessive inflammatory cell infiltration could be apparently found. **f** Results of pathological assessments on knee joint damage for every pathological index including synovial hyperplasia, cartilage damage, vascular proliferation and inflammatory cell infiltration. Data are mean ± SEM (*n* = 15). ^**^ *p* < 0.01 compared with the normal group

Representative photos of non-injected hind paws in normal and AIA rats were taken on day 26 after induction (Fig. 1a, b). AIA rats exhibited an apparent swelling of non-injected hind paws, which was termed as secondary inflammation (Fig. 1b). Statistical results indicated that the secondary paw swelling of AIA rats significantly increased on day 14, 18, 22 and 26 compared with normal rats (Fig. 1c). Histopathological examinations illustrated the severity of knee joint damage (Fig. 1 d, e). There was no inflammation or cartilage damage in knee joint sections from normal rats (Fig. 1d). In contrast, the sections from AIA rats exhibited many pathological features resembling RA such as synovial hyperplasia, cartilage damage, vascular proliferation and inflammatory cells infiltration (Fig. 1e). The individual pathological score for every pathological index in AIA rats was significantly increased compared with that in normal rats (Fig. 1f).

### AQP4 was highly expressed in articular cartilage of AIA rats

**Fig. 2:**
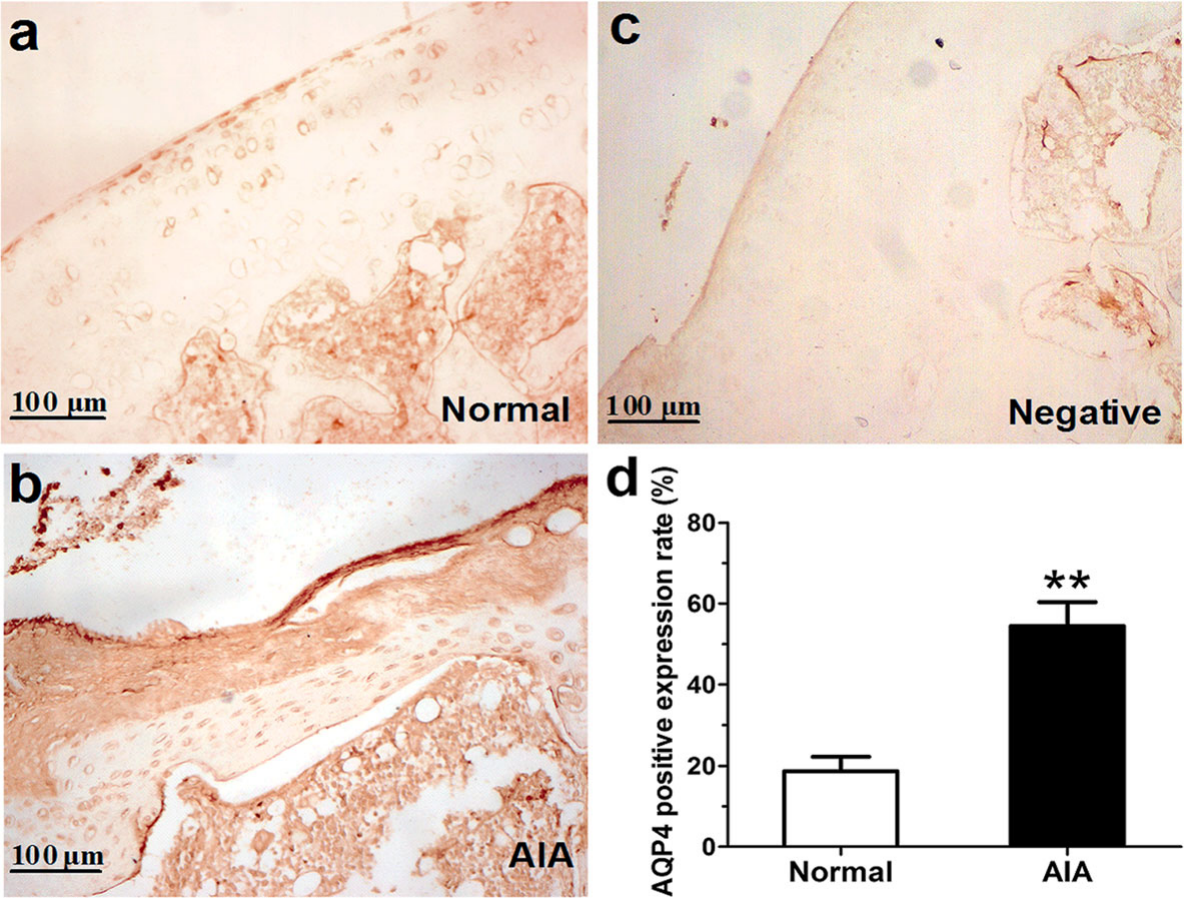
Immunohistochemistry for AQP4 in articular cartilage of knee joints. **a** Normal rat (×200). **b** AIA rat (×200). **c** The negative control was enclosed to show the antibody specificity. **d** AQP4 positive expression rate in articular cartilage of knee joints. The rate is defined as (number of positive cells/total cells) × 100%. Data are mean ± SEM (*n* = 15). ^**^ *p* < 0.01 compared with the normal group

Immunohistochemistry assay was used to investigate the localization and the relative expression levels of AQP4 in articular cartilage of rats. The typical images of AQP4 staining were shown in Fig. 2. AQP4-immunopositive articular chondrocytes in cartilage were visibly more in AIA rats (Fig. 2b) than those in normal rats (Fig. 2a). The Negative control was enclosed to show the antibody specificity (Fig. 2c). The results of statistical analysis on AQP4 positive expression rate in cartilage tissues indicated that AQP4 positive expression rate in AIA rats was dramatically elevated than that in normal rats (Fig. 2d). The semi-quantitative analysis result of AQP4 immunohistochemistry staining in cartilage tissues was shown in Table 1. No strongly (++++) positive expression of AQP4 was found in normal rats, while no negative (−) expression of APQ4 in AIA rats. In addition, the rate of moderately (++, +++) and strongly (++++) positive expression of AQP4 in AIA rats (12/15, 80%) was much higher than normal rats (3/15, 20%). These forementioned findings indicated that AQP4 expression was obviously up-regulated in articular cartilage of AIA rats.

**Table 1:** The semi-quantitative analysis result of AQP4 immunohistochemistry staining in articular cartilage

Group	Grade of AQP4 positive proportion				
	-	+	++	+++	++++
Normal	3	9	2	1	0
AIA	0	3	4	5	3

The positive proportion is graded as follows: (−), < 5%; (+), 5–25%; (++), 26–50%; (+++), 51–75%; (++++), > 75%

**Fig. 3:**
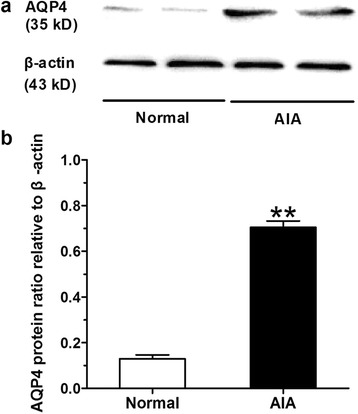
Western blot analyses for AQP4 in cartilage tissues. **a** Representative examples of AQP4 protein expressions in cartilage tissues of normal and AIA rats. **b** Semi-quantitative statistical graph of AQP4 protein levels in cartilage tissues from knee joints of normal and AIA rats, β-actin serves as the house-keeping protein. Data are mean ± SEM (*n* = 15). ^**^ *p* < 0.01 compared with the normal group

We measured AQP4 protein levels in cartilage tissues by western blot and typical examples of AQP4 protein expressions in normal and AIA group were listed in Fig. 3a. There existed obvious changes of AQP4 expression between normal and AIA rats. The AQP4 protein level was quantified densitometrically by ImageJ software and β-actin was served as a house-keeping protein (Fig. 3b). We found that AQP4 protein levels in cartilage tissues from AIA rats were significantly elevated as compared with normal rats.

### Relationships between AQP4 protein levels and secondary paw swelling or pathological scores on joint damage in AIA rats

**Fig. 4:**
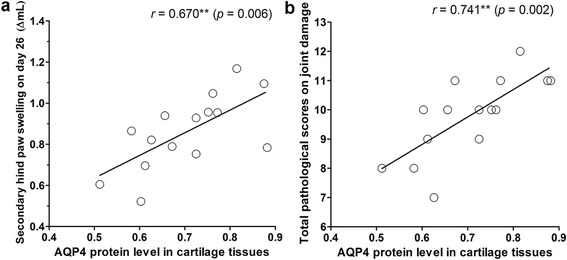
Correlations between AQP4 protein levels in cartilage tissues of AIA rats and secondary paw swelling on day 26 (**a**) or total pathological scores on joint damage (**b**). The correlation analysis was performed by Pearson’s correlation test (*n* = 15). Asterisks (*) show the significance of the correlation. ^**^ *p* < 0.01

AQP4 protein levels in cartilage tissues of AIA rats significantly correlated positively with secondary hind paw swelling on day 26 after induction (Fig. 4a, *r* = 0.670, *p* < 0.01) and total pathological scores on knee joint damage (Fig. 4b, *r* = 0.741, *p* < 0.01). The results of correlation analysis revealed that the elevated AQP4 protein levels in cartilage might be associated with the disease severity of AIA rats.

### Cultured chondrocytes identification and AQP4 immunocytochemistry staining

**Fig. 5:**
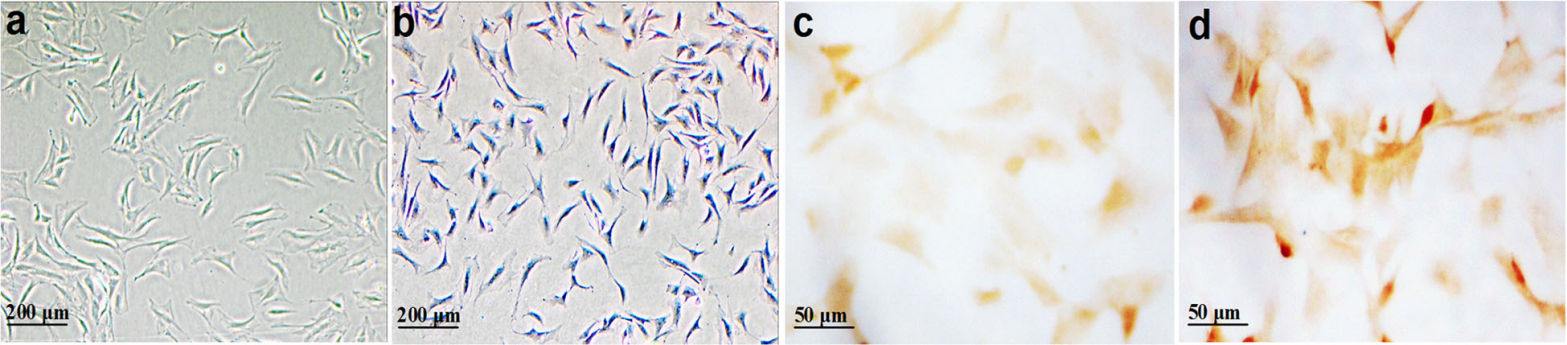
Identification and AQP4 immunocytochemistry staining in cultured articular chondrocytes. **a** Morphology of cultured cells after one passage (×100). **b** Positive toluidine blue staining of glycosaminoglycan in cultured cells (×100). **c** Weak AQP4 immunoreaction in cultured normal articular chondrocytes (×400). **d** Strong AQP4 immunoreaction in cultured AIA articular chondrocytes (×400)

The cultured cells after one passage showed a long spindle-shaped morphology which was accordant with the morphological property of chondrocytes (Fig. 5a). In addition, the cultured cells were identified to be chondrocytes by the positive toluidine blue staining of glycosaminoglycan (Fig. 5b). We detected the expression of AQP4 in cultured normal and AIA articular chondrocytes by immunocytochemistry. Consistent with our findings in vivo, a weak AQP4 immunocytochemistry staining was observed in cultured normal articular chondrocytes (Fig. 5c), whereas a very strong AQP4 immunoreaction in cultured AIA articular chondrocytes (Fig. 5d).

### Acetazolamide inhibited AQP4 protein level in cultured AIA chondrocytes

**Fig. 6:**
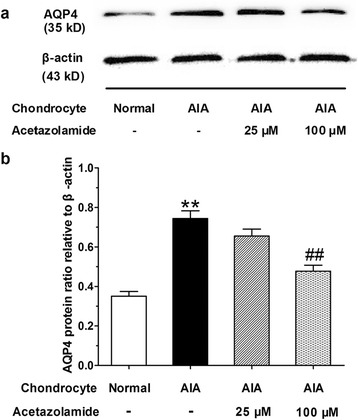
Western blot analyses for AQP4 in cultured articular chondrocytes. **a** Representative examples of AQP4 protein expressions in various cell groups: normal articular chondrocytes, AIA articular chondrocytes, AIA articular chondrocytes treated with acetazolamide (25 or 100 μM). **b** Semi-quantitative statistical graph of AQP4 protein levels in various chondrocytes groups, β-actin serves as the house-keeping protein. Data are mean ± SEM (*n* = 6). ^**^ *p* < 0.01 compared with normal articular chondrocytes group. ^##^ *p* < 0.01 compared with AIA articular chondrocytes group

The AQP4 protein levels in articular chondrocytes from various cell groups were measured by western blot (Fig. 6a) and analyzed statistically (Fig. 6b). Similar to our findings in vivo, AQP4 protein level in cultured AIA articular chondrocytes was higher than that in normal articular chondrocytes (2.1 fold). Acetazolamide treatment reduced the elevated AQP4 protein level in AIA articular chondrocytes. In addition, acetazolamide (100 μM) caused AQP4 protein level to decrease to 63% of the level in AIA articular chondrocytes group.

### Acetazolamide induced cell proliferation of AIA articular chondrocytes

**Fig. 7:**
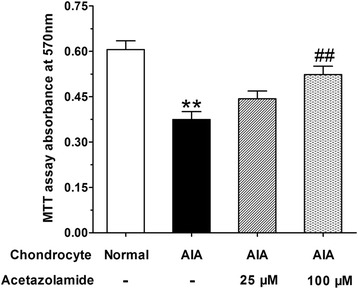
The cell proliferation in various articular chondrocytes groups including normal articular chondrocytes, AIA articular chondrocytes, AIA articular chondrocytes treated with acetazolamide (25 or 100 μM). Data are mean ± SEM (*n* = 6). ^**^ *p* < 0.01 compared with normal articular chondrocytes group. ^##^ *p* < 0.01 compared with AIA articular chondrocytes group

MTT assay was applied to evaluate the potential effect of acetazolamide on cell proliferation of cultured AIA articular chondrocytes. As shown in Fig. 7, the cell proliferation of AIA chondrocytes was lower than that of normal chondrocytes. Acetazolamide (100 μM) treatment produced a significant increase of cell proliferation compared with AIA chondrocytes group, suggesting the pro-proliferative effect of acetazolamide on AIA articular chondrocytes in vitro.

### Acetazolamide increased COII, aggrecan mRNA levels in AIA chondrocytes

**Fig. 8:**
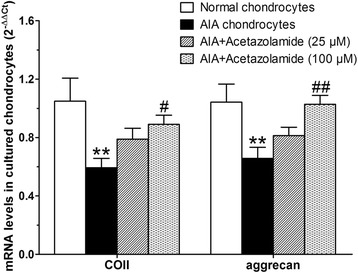
COII and aggrecan mRNA levels in various chondrocytes groups including normal articular chondrocytes, AIA articular chondrocytes and AIA articular chondrocytes treated with acetazolamide (25 or 100 μM). Data are mean ± SEM (*n* = 6). ^**^ *p* < 0.01 compared with normal articular chondrocytes group. ^#^ *p* < 0.05, ^##^ *p* < 0.01 compared with AIA articular chondrocytes group

The mRNA levels of COII and aggrecan in various cell groups were measured by real-time Q-PCR and analysed statistically (Fig. 8). COII and aggrecan mRNA levels in AIA articular chondrocytes were dramatically lower than those in normal articular chondrocytes. Acetazolamide (100 μM) treatment significantly increased mRNA levels of COII and aggrecan compared with AIA articular chondrocytes group, indicating that AQP4 inhibition by acetazolamide could promote ECM production of AIA articular chondrocytes in vitro.

## Discussion

AIA is a classic experimental polyarthritis model resembling RA and shares many pathological features of RA such as extremities swelling, joint inflammation, synovial hyperplasia and cartilage damage [25]. Herein, we utilized rat AIA model to explore the potential pathogenic role of AQP4 over-expression in articular chondrocytes in RA disease progress. There are two stages in AIA progress, i.e., the primary and the secondary inflammation. The primary inflammation stage is characterized by a rapid onset of inflammation in the adjuvant-injected paws within 24 h and gradually lessens after lasting 3 days. Then all paws begin to swell about on day 14 after immunization as a result of the immune response to the bacterial adjuvant, which is called the secondary inflammation stage. The secondary inflammation is the most sensitive parameter in exploring the pathogenesis and the potential pharmacotherapy for RA [26, 27]. Consistent to previous studies [26, 27], in order to avoid potential destructive severe inflammation of the adjuvant-injected paws, we measured the volume changes of the non-injected hind paws at the secondary inflammation stage. In this study, we successfully established AIA rat model by intradermal injection of CFA, evidenced by increased secondary hind paw swelling and aggravated severity of knee joint damage.

Articular chondrocytes are sensitive to extracellular environment changes. In response to ionic and osmotic stress, articular chondrocytes modify the cell shape and cellular organization, which is mediated by cytoskeletal reorganization and cytoplasm membrane transport mechanisms [28]. This biological regulation is essential for ECM homeostasis maintenance and metabolic activity [29]. As we know, AQPs are expressed in a variety of water transporting epithelia and many other tissues where they facilitate water transport across the cell membrane. Since 70% of the total tissue weight in cartilage ECM is water [30], water transport in cartilage ECM and the metabolic water across the membranes of chondrocytes may be important in both normal and pathological conditions of cartilage. Interestingly, previous studies revealed underlying involvement of AQPs (mainly focusing on AQP1 and 3) in cartilage destruction of RA and OA [13–16]. Herein, the present study firstly, to our knowledge, indicated the potential pathologic role of AQP4 over-expression in RA development, as modeled by AIA. The results of immunohistochemistry and western blot indicated that AQP4 protein level was significantly higher in cartilage tissues from AIA rats than normal rats. Particularly, correlation analysis results indicated that the AQP4 protein levels in cartilage tissues from AIA rats correlated positively with the severity of AIA, as determined by secondary hind paw swelling and total pathological scores on knee joint damage. In addition, we isolated and prepared articular chondrocytes from knee joints of rats, as identified by the cell morphology and positively stain of glycosaminoglycan. Consistent with our findings in vivo, AQP4 protein level were significantly elevated in cultured AIA chondrocytes compared with normal chondrocytes. These abovementioned results suggested that AQP4 was over-activated in articular chondrocytes both in vivo and in vitro, and might be closely related to the disease severity and progress of rats AIA.

Recently, acetazolamide is shown to be a potent inhibitor of AQPs and has been extensively applied in many vivo studies, with no obvious adverse effects or serious toxicity [31, 32]. Many studies have revealed that acetazolamide could inhibit AQPs-mediated water permeability in various cell types at the concentrations ranging from 1 μM to 100 μM [20, 21, 33]. Gao J et al. found that acetazolamide (10 and 100 μM) significantly reduced water osmotic permeability in HEK293 cells transfected with pEGFP/AQP1 [20]. Yue Y et al. reported that acetazolamide (100 μM) significantly inhibited AQP1 expression in cultured fibroblast-like synoviocytes of rheumatoid arthritis [33]. Tanimura et al. showed that acetazolamide reversibly suppressed water conduction by AQP4 in a dose-dependent manner and suggested that acetazolamide might be served as a lead compound for the development of AQP4-specific inhibitors [34]. Therefore, acetazolamide was acted as a suitable AQPs inhibitor in the current study. Consistently, we used the concentrations of acetazolamide (25 and 100 μM) in vitro exprements. We found that acetazolamide (100 μM) treatment effectively inhibited the elevated AQP4 protein level in cultured AIA articular chondrocytes.

In arthritis development such as RA and OA, articular chondrocytes damage breaks the balance between biosynthesis and degradation of cartilage ECM and finally causes progressive cartilage damage. Articular cartilage homeostasis is the result of a complex interaction between anabolic and catabolic, anti- and pro-apoptotic, anti- and pro-inflammatory activities. Interestingly, articular chondrocytes death mediated by excessive apoptosis in cartilage has been reported in RA [35, 36] and is considered to be an important and independent factor contributing to RA cartilage degradation [37]. During apoptosis, one of the earliest morphological changes is cell shrinkage known as the apoptotic volume decrease (AVD) [38]. Many studies have suggested that water loss from apoptotic cells during the AVD is primarily mediated by AQPs channels [39, 40]. In particular, inhibition of AQPs-dependent water movement can inhibit the AVD and the subsequent apoptotic events, whereas AQPs over-expression can increase the plasma membrane water permeability and the rate of apoptosis [40]. In this study, cell proliferation of cultured AIA chondrocytes was obviously decreased compared with normal chondrocytes, while acetazolamide treatment effectively increased the cell proliferation of AIA articular chondrocytes. Further studies should be performed to clarify whether the pro-proliferative effect of acetazolamide on AIA chondrocytes is mediated by inhibiting cell apoptosis. It is well known that proteoglycans loss throughout ECM and disturbed collagen fibrils are typical pathologic features of cartilage degradation in RA. COII is the major structural collagen in cartilage and aggrecan forms a major structural component of cartilage together with COII. Herein, we found that acetazolamide treatment reversed the reduced mRNA levels of COII and aggrecan in cultured AIA chondrocytes, suggesting that inhibition of AQP4 by acetazolamide might directly promote ECM production of AIA chondrocytes in vitro.

## Conclusions

Taken together, the current study revealed that AQP4 was overactivated in articular chondrocytes of AIA rats and the elevated AQP4 protein levels in cartilage correlated positively with the severity of AIA. Acetazolamide treatment normalized the dysfunction of AIA chondrocyte in vitro, as indicated by decreasing AQP4 protein level, inducing cell proliferation and increasing mRNA levels of COII and aggrecan. For the first time these findings indicate that AQP4 over-expression might be closely involved in the pathogenesis of RA. Further investigations are needed to observe the therapeutical effect of acetazolamide on AIA and its related molecular mechanisms.

## Abbreviations

AIA: Adjuvant-induced arthritis
AQP4: Aquaporin 4
AVD: Apoptotic volume decrease
CFA: Complete Freund’s adjuvant
COII: Type-II collagen
ECM: Extracellular matrix
FCS: Fetal calf serum
HE: Hematoxylin and eosin
OA: Osteoarthritis
RA: Rheumatoid arthritis
SEM: Standard error of the mean
